# Clonal Spread of *Geomyces destructans* among Bats, Midwestern and Southern United States

**DOI:** 10.3201/eid1805.111711

**Published:** 2012-05

**Authors:** Ping Ren, Katie H. Haman, Lisa A. Last, Sunanda S. Rajkumar, M. Kevin Keel, Vishnu Chaturvedi

**Affiliations:** New York State Department of Health, Albany, New York, USA (P. Ren, S.S. Rajkumar, V. Chaturvedi);; University of Georgia, Athens, Georgia, USA (K.H. Haman, L.A. Last, M.K. Keel);; University at Albany School of Public Health, Albany (V. Chaturvedi)

**Keywords:** Geomyces destructans, white nose syndrome, bats, fungus, letter, midwestern United States, southern United States, pathogen, fungal typing, Chiroptera

**To the Editor:** Bat geomycosis (white nose syndrome) is caused by the psychrophilic fungus *Geomyces destructans*, which has rapidly spread in the United States and Canada since it was first reported from Albany, New York (*1**,**2*). In 2011, a single genotype of *G. destructans* was found in bats with geomycosis in different parts of New York (*3*). The findings raised the possibility of clonal spread of a new pathogen with serious implications for the survival of the affected bat populations (*4*). To provide information for devising conservation measures, we explored whether this emerging infectious disease is caused by a novel pathogen (*5*). To do so, we genotyped *G. destructans* isolates from the midwestern and southern United States.

During 2010 and 2011, a total of 11 cultures of *G. destructans* were isolated and identified: 1 each from Pennsylvania and Ohio, 3 from North Carolina, and 6 from West Virginia (Figure). The cultures came from 8 little brown bats (*Myotis lucifugus*) and 3 tri-colored bats (*Perimyotis subflavus*). Two recent *G. destructans* isolates from New York and 1 *G. pannorum* isolate were included as controls. Genomic DNA was prepared from fungal growth by the conventional glass bead treatment, phenol–chloroform extraction, and ethanol precipitation. PCR amplifications of 8 *G. destructans* gene fragments (*ALR*, *Bpntase*, *DHC1*, *GPHN*, *PCS*, *POB3*, *SRP72*, and *VPS13*) were performed as described (*3*). The amplicons were sequenced and nucleotides were aligned by Sequencher 4.8 (www.genecodes.com); phylogenetic analyses were done using PAUP*4.0 software (www.sinauer.com).

**Figure:**
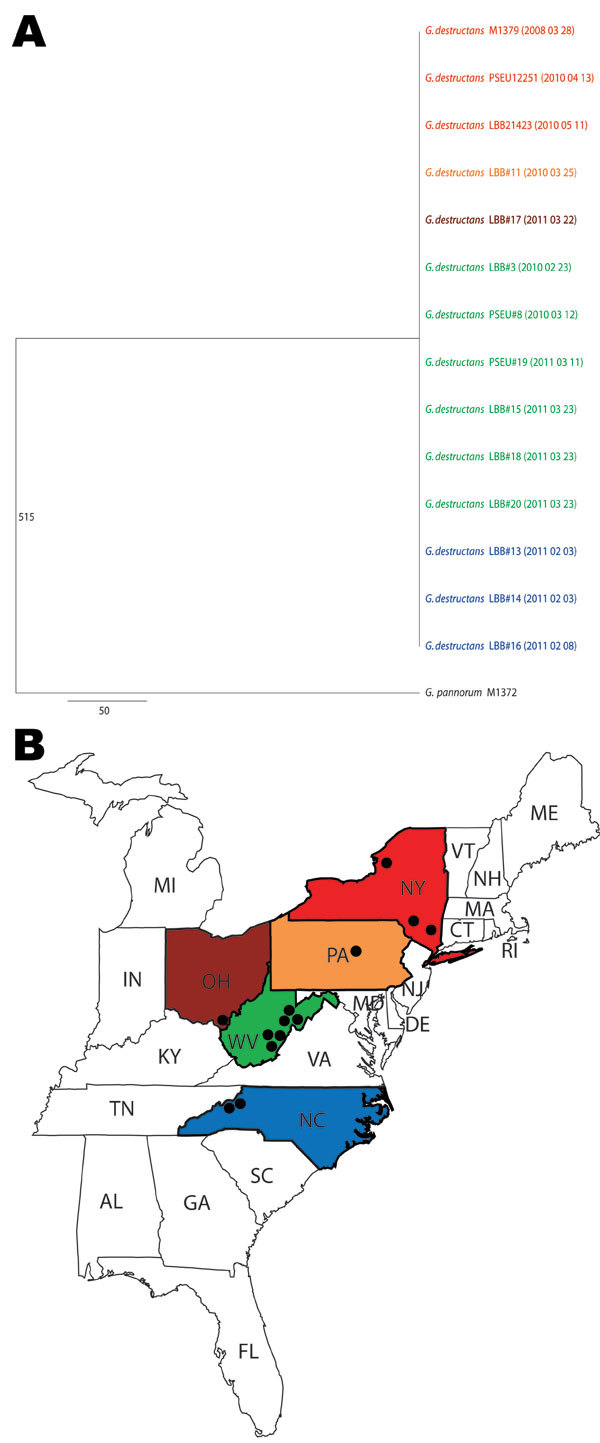
A) Consensus maximum-parsimony tree of 8 concatenated gene fragments of *Geomyces destructans*. Data were derived from 13 *G. destructans* test isolates. *G. destructans* M1379 and *G. pannorum* M1378 were used as controls in this study; they were described in an earlier report (*3*). The number 515 on the branch indicates the total number of variable nucleotide positions (of 4,722 nt) separating *G. pannorum* M1372 from the clonal genotype of *G. destructans*. Isolation dates are shown in parentheses (YYYY MMM DD). Scale bar indicates nucleotide substitutions per site. B) States color-matched to panel A to show where *G. destructans* isolates were found; dots indicate locations of positive test results.

A total of 4,722 nt sequences were obtained from 8 gene fragments of 13 *G. destructans* isolates (GenBank accession nos. JQ029780–JQ029883) and 1 *G. pannorum* isolate (GenBank accession nos. HQ834330, HQ834347, HQ834364, HQ834381, HQ834398, HQ834415, HQ834432, and HQ834449). Multiple alignments of these sequences showed 100% identity, and the aligned nucleotides matched perfectly with those of earlier *G. destructans* sequences for the same gene fragments analyzed from New York isolates (*3*). The nucleotide alignments of 8 sequences showed differences from those obtained from the closely related fungus, *G. pannorum*. Maximum-parsimony trees were generated by using sequences from each gene fragment. These trees showed a single clade of *G. destructans* strains distinct from *G. pannorum*; similar topologies were obtained when different phylogenetics methods were used for analysis (details not shown). A consensus maximum-parsimony tree derived from the 8 concatenated gene fragments also showed a single clade of *G. destructans* isolates from New York and the midwestern and southern United States (Figure).

The data obtained in this study strongly indicate further clonal spread of *G. destructans* from its origin near Albany, New York. The locations in which *G. destructans* was detected in the current study were spread across 5 states, which were >800 miles from Albany. The test isolates were compared with a New York isolate from 2008, which provided a 4-year temporal variation in our sampling. Bats of 2 species were positive for *G. desctructans* in the current samples, and they yielded the same *G. destructans* genotype. Thus, there is evidence for host-independent spread of a single clone of *G. destructans*.

These data would support the novel-pathogen hypothesis for the origin of bat geomycosis (*5*). However, these conclusions are based on limited sampling because isolations of *G. destructans* from affected bats are uncommon. The demonstration of pure fungal culture in the affected animals is still not the standard for geomycosis diagnostics, and most geomycosis is confirmed by bat morphologic appearance or histopathologic examination. Additionally, our phylogenetics analyses were limited to ≈5 kbp of fungal genomes, which could lead to sampling bias (*3*). Ideally, a large number of *G. destructans* isolates, including isolates from Europe, and additional polymorphic markers would be needed to determine the novel or local origin of this pathogen (*6**,**7*).

The environmental factors that led to introduction or reemergence of *G. destructans* in mines and caves remain unknown, and their contribution in the spread of the fungus through air, water, and soil is yet to be determined (*8*). Although no direct evidence has emerged, a role for anthropomorphic activities (occupational or recreational) in this spread is a distinct possibility (*9*). We provide genetic evidence for further spread of a single genotype of *G. destructans* from Albany, New York, to locations in the midwestern and southern United States. Experimental transmission of geomycosis from infected bats to healthy bats by direct contact has recently been confirmed (*10*). Therefore, *G. destructans* might be rapidly spreading along summer and winter migration routes of bats, which present ample opportunities for mixing of healthy and diseased animals.
